# Case report: A case of epidermolysis bullosa complicated with pyloric atresia and a literature review

**DOI:** 10.3389/fped.2023.1098273

**Published:** 2023-03-23

**Authors:** Caiyun Luo, Liucheng Yang, Zhaorong Huang, Yuqian Su, Yi Lu, Daiyue Yu, Mengzhen Zhang, Kai Wu

**Affiliations:** Department of Pediatric Surgery, Zhujiang Hospital, Southern Medical University, Guangzhou, China

**Keywords:** epidermolysis bullosa, pyloric atresia, newborns, molecular characteristics, clinical characteristics, exome sequencing

## Abstract

**Objective:**

This article aims to explore the diagnosis, molecular characteristics, treatment, and prognosis of epidermolysis bullosa with pyloric atresia (EB-PA).

**Methods:**

The clinical manifestations, diagnosis and treatment, and genetic characteristics of a patient with EB-PA admitted to our hospital were analysed. The disease subtypes, concomitant abnormalities, molecular characteristics, and prognosis of patients with EB-PA were summarized by searching the EB-PA-related literature since 2011.

**Results:**

We present a very low birth weight female infant with skin blisters and pyloric obstruction. Exome sequencing revealed heterozygous mutations in the *ITGB4* gene: c.794dupC (p. S265fs*5) and c.2962G > A (p.A988T). This infant was diagnosed with EB-PA. Coverage of the wounds and Penicillin were used to prevent infection, but the patient eventually developed severe sepsis. A literature review was carried out including 49 cases of EB-PA; among these cases, 34 were preterm infants, weighing between 930 and 3,640 g. Of these EB-PA patients, 28 had accompanying malformations, including urinary system malformations and aplasia cutis congenita (ACC). Thirty-two patients identified the subtype of EB-PA, of whom 25 were diagnosed with junctional epidermolysis bullosa (JEB), 6 with epidermolysis bullosa simplex (EBS), and 1 with dystrophic epidermolysis bullosa (DEB). Genetic testing was conducted on 23 patients, of whom 15 carried Integrin Beta-4 (ITGB4) gene mutations and one JEB patient carried an Integrin Alpha-6 (ITGA6) gene mutation; 4 of the 5 EBS patients had Plectin (PLEC) gene mutations, and the other had an ITGB4 mutation. ITGB4 mutation cases involved 29 mutation sites, primarily concentrated in the region encoding the integrin beta subunit; PLEC mutation cases involved 7 mutation sites. Among all cases, 43 underwent pyloric atresia surgery, of whom 24 died postoperatively, and 6 without surgery therapy died within a short period.

**Conclusion:**

EB-PA is a rare genetic disorder characterized by increased skin fragility and PA involving mutations in the ITGB4, PLEC, or ITGA6 genes. EB-PA has a high incidence of complications and mortality, surgery and supportive therapy are currently the most common treatment options.

## 1. Introduction

Epidermolysis bullosa (EB) is a group of inherited bullous diseases with structural abnormalities caused by genetic mutations of various structural proteins in the skin. Due to the increased fragility of the skin mucosa, mild trauma can cause tension bullous and skin erosion. Based on data collected by the U.S. National Epidermolysis Blister Registry from 1986 to 2002, the incidence of EB is approximately 20 per 1 million live births, and the prevalence is approximately 11 per 1 million live births (1). According to the latest International Consensus Meeting on EB, EB is divided into four types: EB simplex (EBS), junctional EB (JEB), dystrophic EB (DEB) and Kindler EB (KEB) (2). EB leading experts proposed an “onion skin” approach to classification, which further subclassified the four main types of EB into other subtypes by considering their phenotype (severity and distribution), mode of transmission, ultrastructural site of cleavage and associated findings, proteins involved (with or without specific immunofluorescence mapping findings listed), genes involved and mutational type, and specific mutation present (3).

Epidermolysis bullosa with pyloric atresia (EB-PA) is a rare clinical subtype of epidermolysis bullosa (EB) accompanied by high mortality. The diagnosis and management of EB-PA are difficult (4). In this study, we reviewed the clinical data of an infant admitted to our hospital and comprehensively analysed the characteristics of the cases in the literature to summarize the experience in diagnosing and treating EB-PA.

## 2. Case report

**Figure 1:**
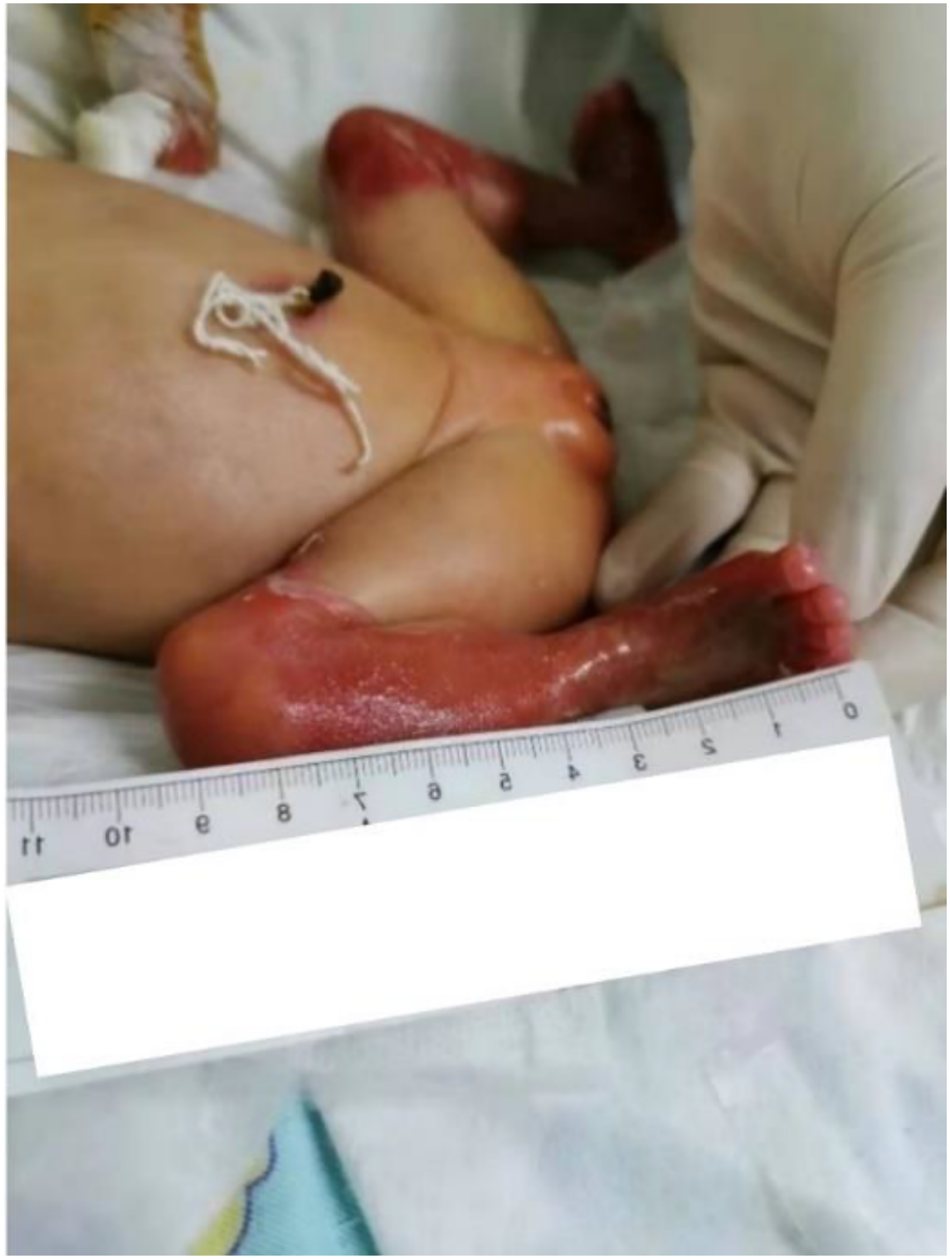
Severe ruptured blisters with skin lesions on both lower limbs.

**Figure 2:**
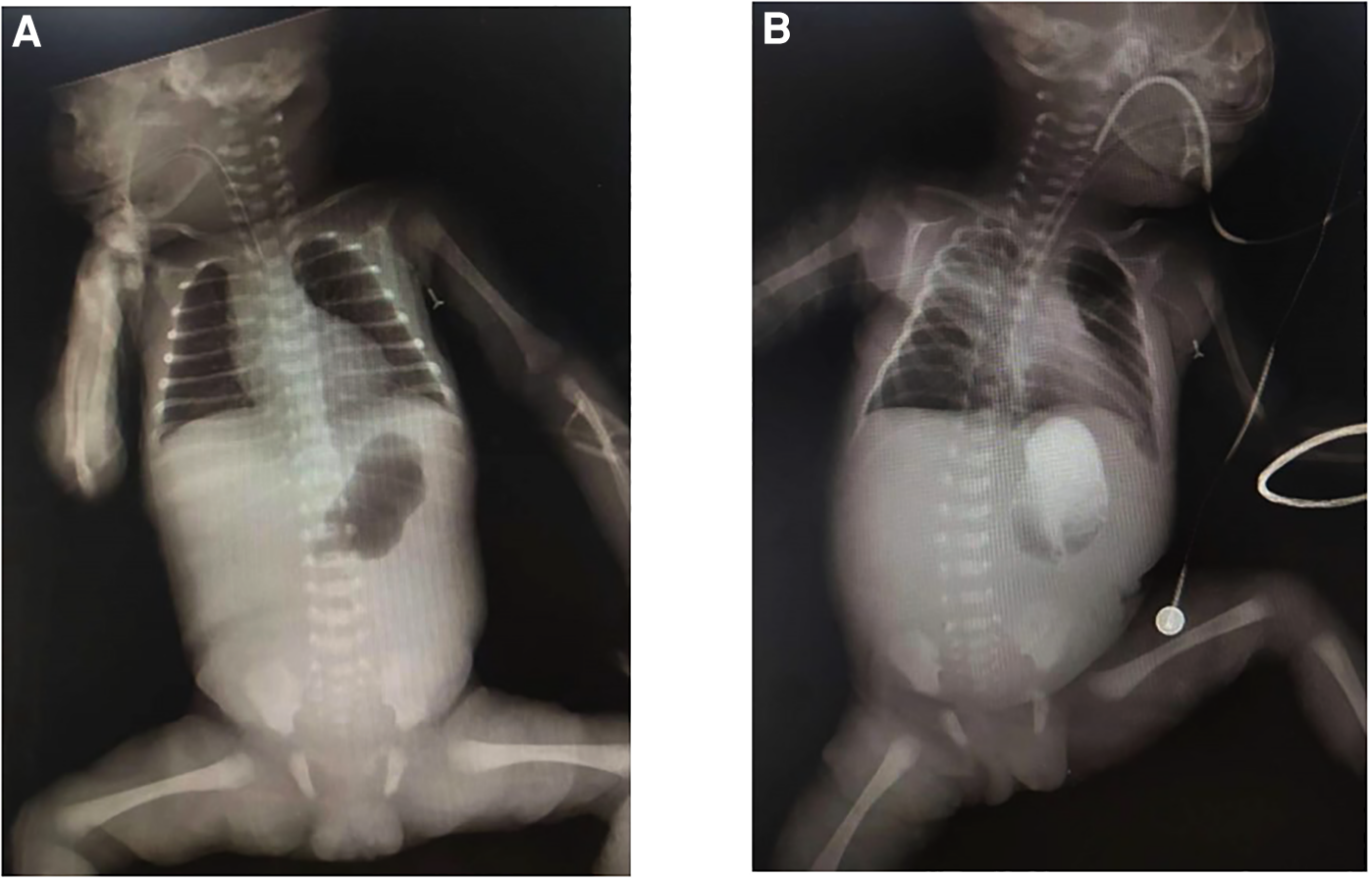
(**A**) x-ray demonstrates a single bubble sign; (**B**) upper gastrointestinal angiography shows no contrast uptake beyond the stomach.

A female preterm infant, the first child of healthy nonconsanguineous parents, was born at 30 + 2 weeks of gestation by normal delivery, with a birth weight of 1,210 g and a negative family history. After birth, multiple skin defects were found in the whole body, mainly in the lower limbs. Physical examination at birth indicated skin defects of both lower limbs with basal flushing and exudation, but no obvious signs of infection were found in the wounds (Figure 1). The dorsum of the foot was a hyperflexion deformity with low muscle tone in the extremities. The upper abdomen was distended with a distinct gastric pattern. Routine blood test was measured and revealed that white blood cell count (WBC) 10.53 × 10^9^/L (reference range 15.0–20.0 × 10^9^/L), neutrophil numerals (Neut#) 8.13 × 10^9^/L (ref. 1.8–6.3 × 10^9^/L), haemoglobin (Hb) 137 g/L (ref. 170–200 g/L), platelets (PLT) 230 × 10^9^/L (ref. 125–350 × 10^9^/L). The results of coagulation function showed prothrombin time (PT) 16.0 s (ref. 9.8–12.1 s), active partial thromboplastin time (APTT) 110.2 s (ref. 25.0–31.3 s), international normalized ratio (INR) 1.38 (ref. 0.88–1.08). x-ray showed gas in the stomach but no inflation in the small intestine, which is considered pyloric obstruction (Figure 2A). Contrast medium accumulation in the stomach lumen was observed on upper gastrointestinal angiography, with no development in the duodenum or distal part (Figure 2B). There were no abnormalities in the respiratory, cardiac or urinary systems. A skin biopsy was recommended to further confirm the diagnosis of EB, but her parents refused to perform this test.

The patient's clinical manifestations and examinations indicated a genetic disease. After obtaining consent from the patient's family, we performed exome sequencing, which revealed heterozygous mutations in the affected *ITGB4* gene, c.794dupC (p. S265fs*5) and c.2962G > A (p. A988T) (Table 1). These two variants were inherited from her parents respectively. The mutation c.794dupC is a frameshift mutation that results in the early termination of the amino acid code for protein synthesis. Another mutation c.2962G > A causes the substitution of a conserved amino acid. Computer analysis predicts that this mutation may affect protein structure and function.

**Table 1:** Genotype with candidate gene abnormalities in this case.

Gene	OMIMID	HG19	NMID	cDNA	Protein	Exon	Mutation type	Genotype	Origin	ACMG
ITGB4	147557	Chr17:73726377	NM_000213	c.794dupC	p.S265fs*5	exon 8	frame-shift	Het	Father	LP
ITGB4	147557	Chr17:73738842	NM_000213	c.2962G > A	p.A988T	exon26	missense	Het	Mother	VUS

Het, heterozygote; LP, likely pathogenic; VUS, variant of unknown significance.

In terms of treatment, sedation was given to reduce skin friction, and measures were taken to keep the skin clean and dry. Recombinant human epidermal growth factor gel was applied to the skin and covered with Vaseline gauze. Penicillin was also used to prevent infection. On the fourth day after admission, the baby began to develop a fever, and the result of routine blood tests showed WBC 1.89 × 10^9^/L, Neut# 0.4 × 10^9^/L, Hb 126 g/L, and PLT 127 × 10^9^/L, while coagulation function revealed PT 18.7 s, APTT 70.6 s, INR 1.61. Meropenem, red blood cells and plasma were transfused, but the infection was difficult to control. The wound exudate increased and tended to bleed when touched. Abdominal distention also worsened. The patient was diagnosed with sepsis. The baby's parents refused further treatment because of the complexity of the disease, and the patient died shortly after being discharged from our hospital.

## 3. Literature review

**Figure 3:**
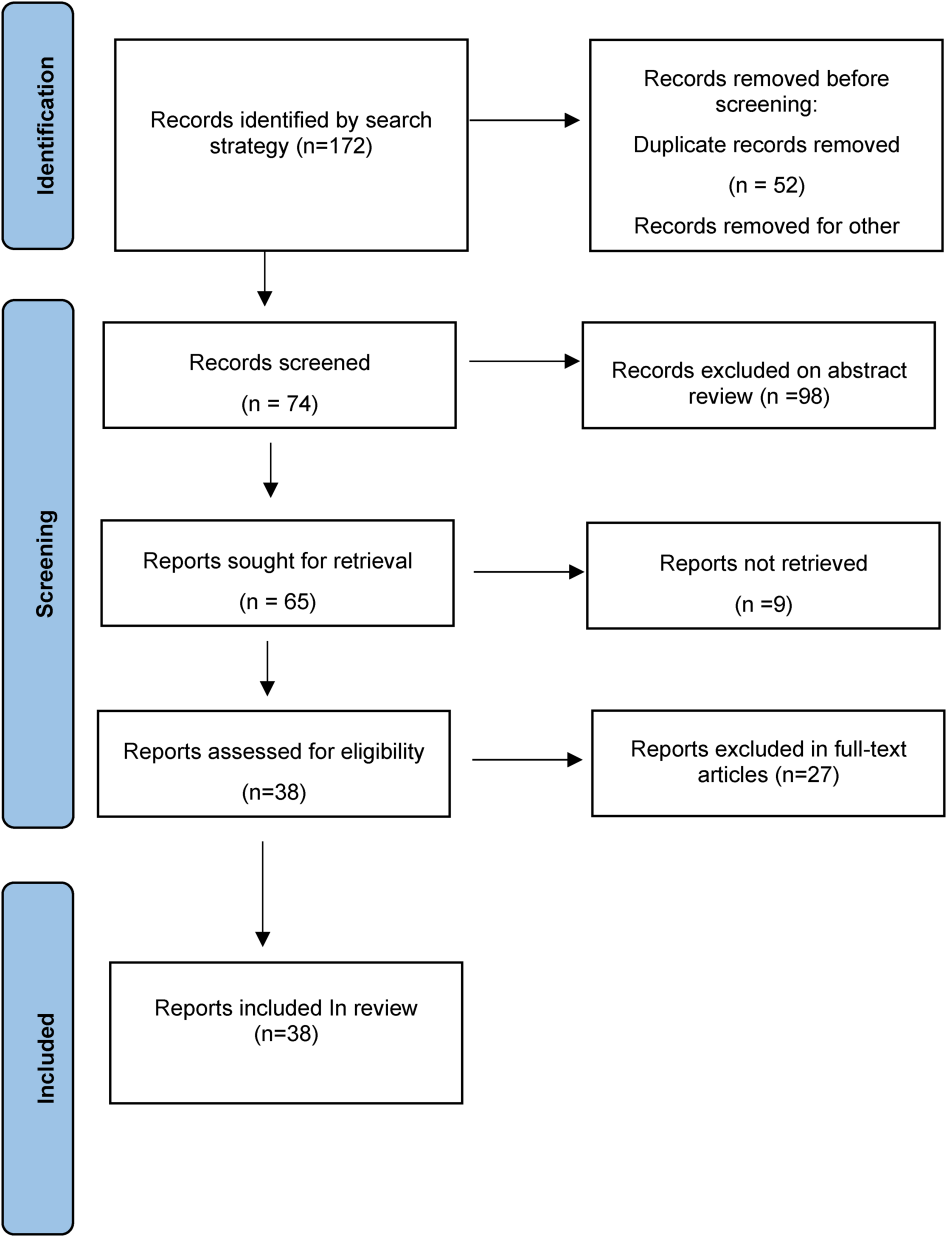
PRISMA flow diagram of the study selection.

Setting the search terms for pyloric atresia and epidermolysis bullosa, the subjects for 0–18-year-old patients, and the publication time from 2011 to 2021, a fuzzy search was conducted in the China National Knowledge Infrastructure (CNKI), PubMed, SinoMed, and Embase databases. According to the content of the full text and the correlation degree of EB-PA disease, 38 articles (2 Chinese articles and 36 English articles) were finally included, with a total of 49 EB-PA cases (Figure 3, Supplementary Table S1) (5–42).

### 3.1. Clinical characteristics

Among these 49 EB-PA cases, the gestational age at birth was documented in 46 patients, except for 3 cases whose gestational age at birth was unknown. Thirty-four were preterm, and the rest were full-term. Birth weights were reported in 28 cases, ranging from 930 to 3,640 g. A total of 28 cases recorded the results of the maternal prenatal examination, including 20 cases with hydramnios, 7 cases with upper gastrointestinal obstruction manifestations such as foetal gastric dilatation on prenatal ultrasound or foetal MRI, and 2 cases with EB typical signs such as the “snowflake sign” of amniotic fluid and chorioamniotic membrane separation on prenatal ultrasound. In addition to skin bullae and pyloric atresia, EB-PA can also be associated with other systemic malformations, especially urinary system lesions and Aplasia cutis congenita (ACC). There were 12 patients with ACC and 13 patients with urinary tract disease, mainly manifesting as polycystic kidney disease and ureter hydronephrosis. Eleven patients had hypoplasia of the outer ear, shortened nose or other abnormal facial features. Ten developed nail malnutrition.

### 3.2. Diagnostic methods and pathological classification

EB-PA subtypes were determined by skin biopsy, transmission electron microscopy (TEM) or immunofluorescence mapping (IFM). Thirty-two patients underwent one or more of the above examinations. Thirty-two cases of specific EB types were mentioned in the literature, including 6 cases of EBS, 25 cases of JEB and 1 case of DEB. There is also a case according to the result of immunohistochemical, is unable to judge EBS or JEB.

### 3.3. Molecular characteristics

**Figure 4:**
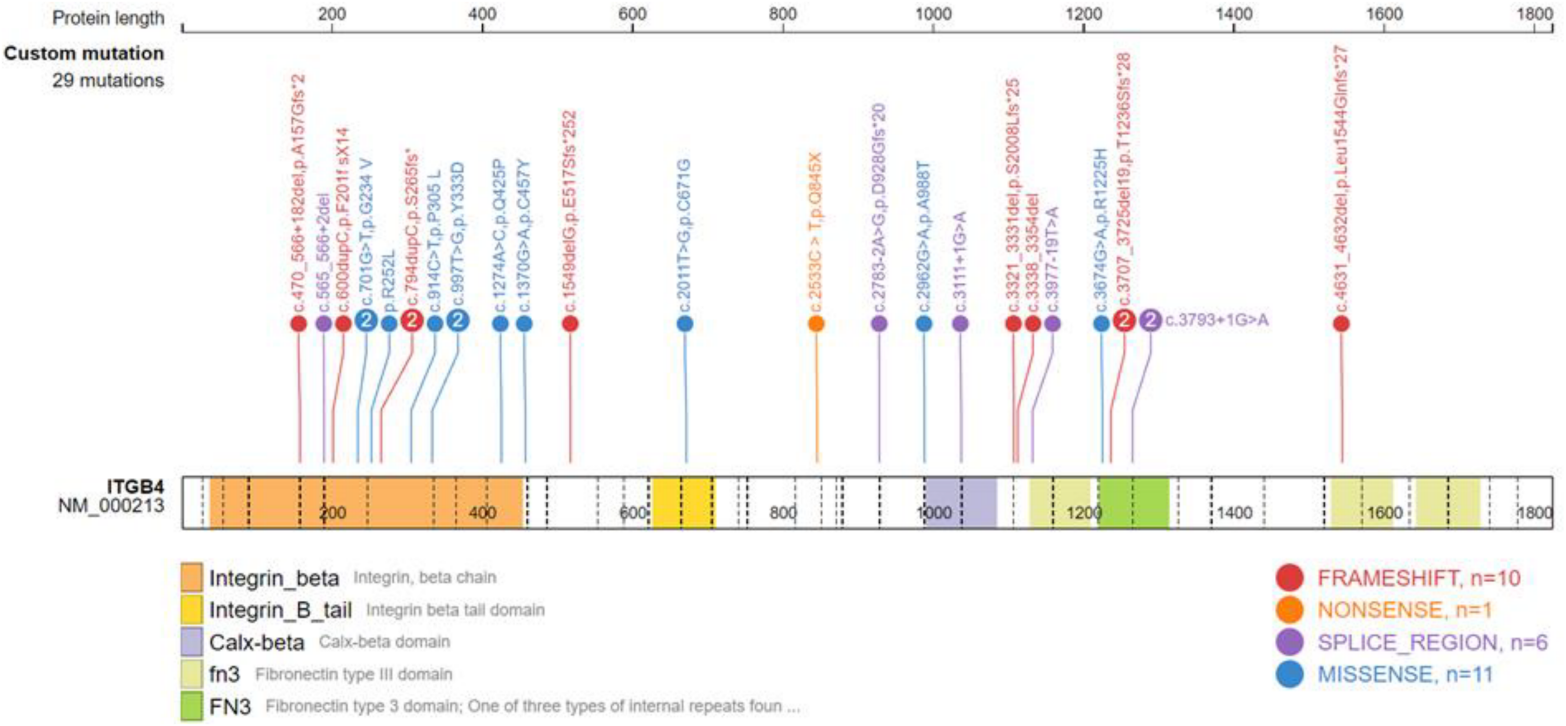
Schematic diagram of the ITGB4 domain and the location of the ITGB4 mutation.

**Figure 5:**
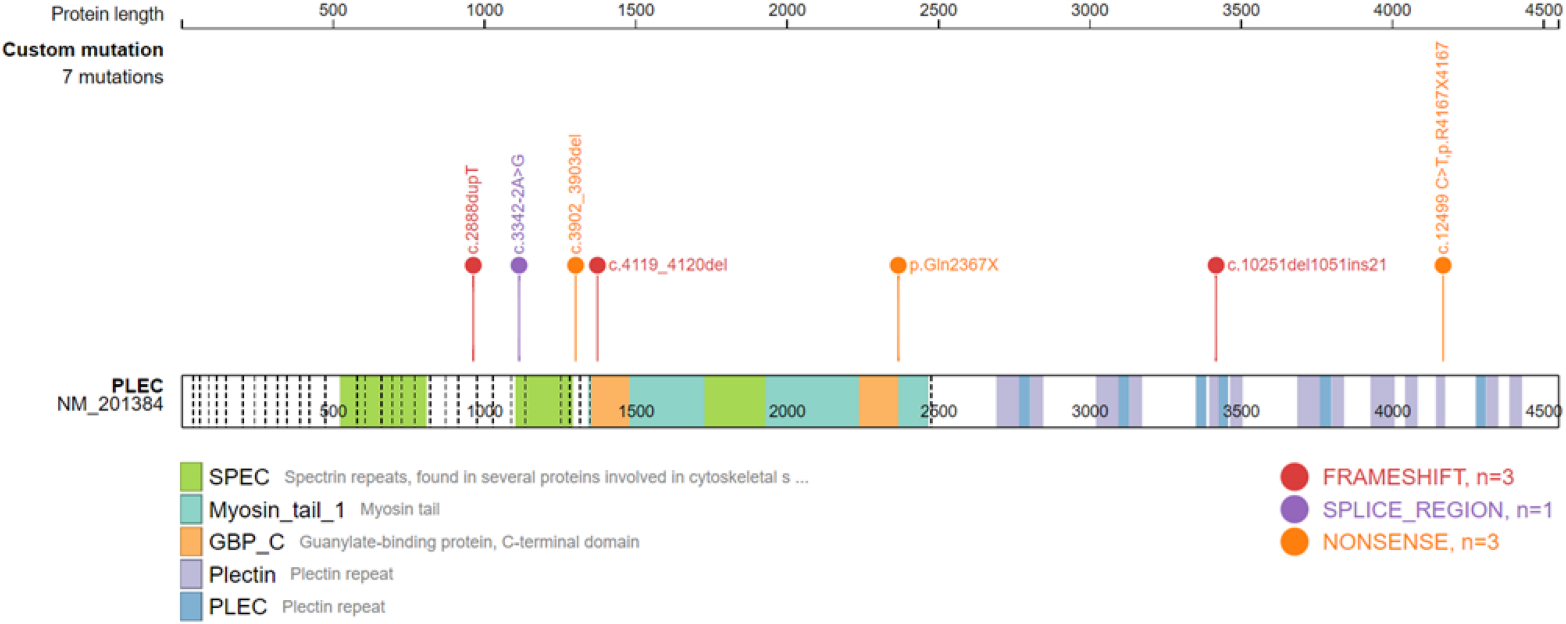
Schematic diagram of the PLEC domain and the location of the PLEC mutatio.

Gene testing was performed in 24 patients, including 17 ITGB4 gene mutation cases. Among the ITGB4 gene mutation cases, 15 were JEB, 1 was EBS, and 1 was of an unknown type. There were 5 PLEC gene mutation cases, 4 of which were diagnosed as EBS, and the last was unclassified. Only one patient with JEB carried the ITGA6 gene mutation. Twenty-seven mutation positions of ITGB4 gene were detected, with 5 duplicated sites, *c.701G > T*, *c.997T > G*, *c.3707-3725del19* and *c.3793 + 1G > A,* and the rest were sporadic (Supplementary Table S2). ITGB4 gene mutation sites were widely distributed, resulting in amino acid substitutions or a premature termination codon. A total of 7 PLEC gene mutation sites were detected in 4 patients (Supplementary Table S3), mainly concentrated in the exon 23–32 region, resulting in premature translation termination. Analysis of the ITGB4 gene and PLEC gene mutation sites using the gene mutation visualization program ProteinPaint (43) revealed that the ITGB4 mutation region was dominated by mutations in the integrin beta domain, with a total of 12 mutation sites located in this region, while the PLEC gene mutation position was mainly located in pletin and spectrin repeats (Figures 4, 5).

### 3.4. Treatment and prognosis

Forty-three patients underwent surgery for PA. Twenty-four of them died postoperatively, with the age of death varying from 5 days to 9 months, and death was related to sepsis or organ failure. Two patients abandoned treatment due to critical postoperative conditions. Five patients did not have postoperative follow-up mentioned. Twelve patients were followed up, ranging from 8 months to 36 years, and 4 of them were without symptoms. Three had mild blistering episodes, and the remainder suffered from urinary system diseases such as cystitis, hydronephrosis, or respiratory tract damage. All patients who did not receive surgical treatment died beyond a month.

## 4. Discussion

By summarizing the EB-PA-related cases published in the past 10 years, we found that the subtypes of EB-PA are basically EBS-PA and JEB-PA. The JEB subtype accounted for 78% of patients for whom the EB subtype had been identified. These are consistent with the international consensus. In addition, there has been a case report of DEB combined with PA (17), which is extremely rare.

Among the 49 EB-PA cases, including ours, 34 were preterm, and 22 weighed less than 2,500 g, which means that most cases of EB-PA are premature low-body weight infants. Abnormalities other than skin blisters and PA were reported in 28 patients, with urinary abnormalities being the most common malformation (13/28), followed by ACC (12/28), ear, nose and throat deformities (11/28), nail malnutrition (10/28) and respiratory tract involvement (5/28). ACC often occurs in conjunction with JEB-PA. Kayki et al. (28) speculated that ACC may be caused by the intrauterine progression of JEB-PA. Urinary system involvement of EB-PA generally manifests as renal dysplasia, such as polycystic kidney, hydronephrosis of the renal pelvis or ureter, acute tubular necrosis, urethral obstruction, ureteral cyst, kidney duplication and agenesis of the bladder (28). If the respiratory system is involved, granulation tissue formation, glottis and subhilar stenosis may arise (44). In 2010, Aydin et al. (45) first reported a child with EB-PA accompanied by partial abnormal pulmonary vein reflux (PAPVR), secondary atrial septal defect (ASD) and pulmonary hypertension, but the correlation between an abnormal circulatory system and EB-PA remains to be proven. Our case was also a low-weight preterm infant, but no concomitants other than EB-PA have been found so far.

Prenatal examination during pregnancy can help to identify a foetus with EB-PA. In our study, most of the infants showed polyhydramnios, amniotic fluid snowflake sign, or chorioamniotic membrane separation on prenatal ultrasound. The concentrations of maternal serum a-fetoprotein (AFP) and amniotic fluid AFP and acetylcholinesterase (AchE) levels are increased in some pregnant women (20, 46). Mutational analysis of fetal DNA obtained from chorionic villus sampling at 10–12 weeks of gestation or amniocentesis at 16 weeks of gestation has also been suggested (47). The postnatal diagnosis of patients with EB-PA relies on the presence of blisters, clinical manifestations of upper gastrointestinal obstruction, and various auxiliary examination methods. The definition of the subtype is necessary, mainly by immunofluorescence antigen mapping (IFM) or transmission electron microscopy (TEM) of skin biopsy tissues to identify the level of blister formation (3). However, many infants did not undergo TEM or IFM. Nine patients underwent only TEM, 10 patients underwent IFM, and 4 patients underwent both TEM and IFM.

EB-PA is commonly inherited in an autosomal recessive manner, and its pathogenesis is complex. In 1995, Vidal et al. (48) first reported that the occurrence of JEB-PA was associated with the deletion or pathogenic variation of integrin α6β4, a key protein of keratinocyte hemidesmosomes. Integrin α6β4 stabilizes the skin basement membrane by connecting laminin-332 to the dense layer beneath the basement membrane. We summarized the mutation sites of the ITGB4 gene with literature review as well as our own cases and found that 12 mutation sites were situated in the region of exons 6–15, which encode the integrin beta domain. Nine sites were located in exons 21–34, encoding the Calx-beta domain and Fibronectin type III or 3 domain. Mutations in these regions caused amino acid substitutions or premature termination of codons, resulting in reduced or absent integrin α6β4 expression. In 2005, Nakamura et al. (49) demonstrated that EBS-PA was associated with mutations in the PLEC gene located in the telomere region of the long arm of chromosome 8. Later, through gene mutation analysis, Winter et al. (50) and Charlesworth et al. (15) found that when the PLEC mutation in EBS-PA patients was located outside exon 31 or its C-terminal part, the expression of full-length and rodless plectin isoforms was significantly reduced or completely lost, thus causing EB-PA. In our study, 2 mutation sites were located in exon 32, and 1 was located in exon 31. All these mutations caused premature termination codon or splicing abnormalities. EB-PA patients with mutations in ITGA6 or PLEC genes have a high death rate. The prognosis is often poor if ITGB4 mutations are located in highly conserved or protein-binding regions (51).

The early improvement of skin lesions in patients and prevention, monitoring and treatment of complications are the main goals of the treatment of EB (52). Treatment measures for skin lesions are to avoid mechanical stress and to take good care of the skin to prevent further damage caused by skin friction. The wound can be covered with adhesive-free dressings that absorb exudate, and appropriate sedative and analgesic drugs, antihistamine receptors and other drugs can be used to reduce the symptoms of pain and itch in patients (4). In cases of secondary wound infection, antibiotics should be selected according to the results of bacterial culture and drug sensitivity tests (53). In our case, considering that the patient's lesions were too extensive and the risk of infection was high, penicillin was used prophylactically. However, on the fourth day after birth, the infection occurred despite careful care of the wound. The infection was difficult to control and eventually progressed to sepsis. In the literature review, 2 patients also died of sepsis without surgery. Hayashi et al. (54) suggested that surgical therapy of PA should be performed after the skin lesions are stabilized, which could appropriately prolong the survival time of patients. Pyloroplasty is feasible for type I and type III PA, while end-to-side or side-to-side gastroduodenostomy is required for type II PA (13). Even if PA is successfully treated by surgery, postoperative patients may die from postoperative complications such as infection, severe electrolyte imbalance, intestinal obstruction, malnutrition, and hypostatic pneumonia (5). The majority of infants in our study were treated surgically, and more than half died from complications such as sepsis, organ failure, and water-electrolyte imbalance. The postoperative mortality and complications of EB-PA were extremely high. The few surviving cases were mostly infants with mild skin lesions, but there was still a risk of repeated infection and a poor prognosis. In recent years, protein, gene or cell replacement therapy and allogeneic bone marrow transplantation have been successfully carried out in animal experiments, which are expected to be put into clinical practice (4).

In conclusion, the diagnosis and treatment of EB-PA is our current challenge. Its treatment requires multidisciplinary medical, nursing, psychological and social management (55). Even if the disease is currently improving, there is still a risk of blistering recurrence, and long-term follow-up is necessary.

## Data Availability

The original contributions presented in the study are included in the article/**Supplementary Material**, further inquiries can be directed to the corresponding author.
