# Supplementary material for: Case report: A case of epidermolysis bullosa complicated with pyloric atresia and a literature review
Source: Front Pediatr. 2023 Mar 23;11:1098273. doi: 10.3389/fped.2023.1098273 (PMC10076629; doi:10.3389/fped.2023.1098273)
Supplement: Supplementary file 1 [file Datasheet1.pdf]

Table 1 Review of EB-PA cases in the last decade

case	Reference (year)	Age at birth	Age at death	Birth weight (g)	EB type	Diagnose methods				Mutant gene	Concomitant Anomalies					surgery	sepsis
						SB	IFM	TEM	GT		US	RS	ACC	ENT	Nail		
1	Kim(5), 2011	33 w	NM	2330	JEB	+	+	+	-	NM	-	-	-	-	-	+	-
2	Bicakci(6) , 2012	33 w	2 m	2000	NM	-	-	-	-	NM	-	-	-	-	-	+	+
3	Bicakci(6) , 2012	35 w	25 d	2200	NM	-	-	-	-	NM	-	-	-	-	-	+	+
4	Lichtenstein(7), 2012	31 w	35d	NM	EBS	+	-	+	-	NM	-	-	-	+	-	+	+
5	Lichtenstein(7), 2012	full-term	24 d	NM	EBS	+	-	+	+	PLEC	-	-	-	+	-	-	-
6	Stoevesandt(8), 2012	26^{+4}w	18 d	930	JEB	+	+	-	+	ITGB4	-	-	-	+	-	+	+
7	Diociaiuti(9), 2012	31 w	alive	1600	JEB	+	+	+	+	ITGB4	-	-	-	-	+	+	-
8	Fu(10), 2013	full-term	alive	1820	NM	+	-	-	-	NM	-	-	-	-	-	+	-
9	Merrow(11), 2013	35 w	18 d	NM	NM	+	+	-	+	PLEC	-	-	+	+	-	+	+
10	Mithwani(12), 2013	33 w	25 d	1480	NM	+	-	-	-	NM	-	-	-	-	-	+	+
11	Son(13), 2013	full-term	NM	NM	NM	-	-	-	-	NM	-	-	-	-	-	+	-
12	Marjanovic(14), 2013	36 w	9 mon	2900	JEB	+	-	-	-	NM	-	-	-	-	-	+	+
13	Charlesworth(15), 2013	33 w	1 d	NM	EBS	-	-	-	+	PLEC	+	-	+	+	-	-	-
14	Charlesworth(15), 2013	NM	alive	NM	EBS	-	-	-	+	PLEC	-	-	-	-	-	+	-
15	Hassan(16), 2013	37 w	6 mon	2840	JEB	+	-	+	-	NM	-	+	+	-	+	+	-
16	Short(17), 2014	29 w	NM	1200	DEB	+	-	+	-	NM	-	-	-	-	-	+	+
17	Hon(18), 2014	full-term	4 mon	1820	EBS? JEB?	+	-	-	-	NM	-	-	-	-	-	+	+
18	Farmakis(19), 2014	31 w	alive	1275	JEB	+	-	-	-	NM	+	-	-	-	-	+	-
19	Dural(20), 2014	35 w	alive	NM	NM	-	-	-	-	NM	-	-	-	-	-	+	-
20	Dural(20), 2014	33^{+5}w	12 d	1450	NM	-	-	-	-	NM	-	-	-	-	-	+	+
21	Joshi(21), 2014	32 w	12 d	NM	NM	-	-	-	-	NM	-	-	-	-	-	+	+
22	Schutzman(22), 2014	33 w	3 mon	1290	JEB	+	-	+	-	NM	-	-	-	-	-	+	-
23	Chahed(23), 2015	full-term	4 mon	2350	NM	+	-	-	-	NM	-	-	-	-	-	+	-
24	Chahed(23), 2015	full-term	3 mon	3100	NM	-	-	-	-	NM	-	-	-	-	-	+	-
25	Yang(24), 2016	full-term	alive	3000	NM	-	-	-	-	NM	-	-	-	-	-	+	-
26	Mencia(25), 2016	31 w	alive	NM	EBS	+	+	-	+	ITGB4	-	-	-	-	+	+	-
27	Mencia(25), 2016	32 w	alive	NM	JEB	+	+	+	+	ITGB4	+	-	-	-	+	+	-
28	Mencia(25), 2016	36 w	20 d	NM	JEB	+	+	-	+	ITGB4	+	-	+	-	-	+	-
29	Mencia(25), 2016	35 w	33 d	NM	JEB	+	+	-	+	ITGB4	-	-	+	-	+	+	-
30	Mencia(25), 2016	34 w	17 d	NM	JEB	+	+	-	+	ITGB4	+	-	+	-	-	+	+
31	Mencia(25), 2016	34 w	6 mon	NM	JEB	+	+	-	+	ITGB4	-	-	+	-	-	+	-
32	Walker(26), 2017	34 w	alive	NM	EBS	+	-	-	+	PLEC	+	+	-	-	-	+	-

33	Mitra(27), 2017	premature	alive	NM	NM	-	-	-	-	NM	+	+	-	-	-	+	-
34	Kayki(28), 2017	full-term	33 d	2450	JEB	+	-	+	+	ITGB4	+	-	+	-	+	+	-
35	Fu(29), 2017	38^{+1}w	alive	3640	NM	-	-	-	+	NM	-	-	-	-	-	+	-
36	Ko(30), 2018	35^{+6}w	alive	2720	JEB	+	-	+	+	ITGB4	-	-	-	-	-	+	-
37	Hattori(31), 2018	37 w	alive	2350	NM	-	-	-	+	ITGB4	-	-	-	-	+	+	-
38	Trah(32), 2018	34 w	41 d	1780	JEB	+	+	+	-	NM	-	-	+	-	-	+	-
39	Hicks(33), 2018	30 w	17 d	NM	JEB	+	+	-	-	NM	+	+	+	+	-	-	-
40	Al(34), 2020	33 w	11 d	2000	NM	-	-	-	-	NM	-	-	-	+	-	-	+
41	Okulu(35), 2020	31 w	4 d	1620	JEB	+	-	-	+	ITGB4	+	-	-	-	+	-	-
42	Verma(36), 2020	NM	alive	NM	JEB	+	-	+	+	ITGB4	+	-	-	+	+	+	-
43	Schreiner(37), 2021	32^{+1}w	35 d	1400	JEB	+	+	-	+	ITGA6	+	-	+	+	-	+	-
44	Matyas(38), 2021	32 w	5 d	NM	JEB	-	-	-	+	ITGB4, KRT10	-	-	+	+	+	+	-
45	Wee(39), 2021	36 w	alive	1500	JEB	+	+	-	+	ITGB4	-	+	-	-	-	+	-
46	Chen(40), 2021	premature	1 mon	NM	JEB	-	-	-	-	NM	-	-	-	+	-	-	+
47	Chong(41), 2021	full-term	4 mon	1820	JEB	+	-	+	-	NM	-	-	-	-	-	+	+
48	Chong(41), 2021	35^{+3} w	3 mon	2330	JEB	+	-	-	+	ITGB4	-	-	-	-	-	+	+
49	Ellis(42), 2021	NM	alive	NM	JEB	-	-	-	+	ITGB4	+	-	-	-	-	+	-

EBS, epidermolysis bullosa simplex; JEB, junctional epidermolysis bullosa; DEB, dystrophic epidermolysis bullosa; SB, skin biopsy; IFM, immunofluorescence mapping; TEM, transmission electron microscopy; US, Urinary system; RS, Respiratory system; ENT, ear nose throat; ACC, aplasia cutis congenita; NM, not mentioned; +, yes; -, no

Table 2 Mutation sites and mutation types related to the ITGB4 gene

Case	Reference (year)	Mutation	Protein change	Position	Mutation type
6	Stoevesandt(8),2012	c.600dupC	p.F201f sX14, PTC	exon7	F
		c.2533C > T	p.Q845X, PTC	exon21	N
7	Diociaiuti(9),2012	c.3338_3354del	PTC	exon 28	F
		c.3977-19T>A	--	intron 31	P
26	Mencia(25), 2016	c.3674G>A	p.R1225H	exon30	M
27	Mencia(25), 2016	c.997T>G	p.Y333D	exon8	M
		c.1370G>A	p.C457Y	exon11	M
28	Mencia(25), 2016	c.470_566+182del	p.A157Gfs*2	exon6	F
		c.2783-2A>G	p.D928Gfs*20	intron24	P
29	Mencia(25), 2016	c.997T>G	p.Y333D	exon8	M
		c.3321_3331del	p.S2008Lfs*25	exon28	F
30	Mencia(25), 2016	c.701G>T	p.G234 V	exon7	M
		c.3707_3725del19	p.T1236Sfs*28	exon30	F
31	Mencia(25), 2016	C.701G>T	p.G234 V	exon7	M
		c.3707_3725del19	p.T1236Sfs*28	exon30	F
34	Kayki(28),2017	c.3793+1G>A	--	intron 30	P
36	Ko(30), 2018,	c.914C>T	p.P305 L	exon8	M
		c.2011T>G	p.C671G	exon15	M
37	Hattori(31), 2018,	c.1274A>C	p.Q425P	exon11	M
		c.1549delG	p.E517Sfs*252	exon13	F
41	Okulu(35), 2020	c.565_566+2del	PTC	exon 6/intron 6	P
42	Verma(36),2020	c.4631_4632del	p.Leu1544Glnfs*27	exon 34	F
44	Matyas(38), 2021	c.3111+1G>A	--	exon26	P
45	Wee(39), 2021	c.794dupC	p.S265fs*5	exon8	F
		c.1608C>T	p.Cys536Cys	exon13	S
49	Ellis(42),2021	c.3793+1G>A	--	intron30	P
		--	p.R252L	exon8	M
50	our case	c.794dupC	p.S265Sfs*5	exon 8	F
		c.2962G>A	p.A988T	exon 26	M

PTC, premature termination codon; F, frame-shift mutation; N, nonsense mutation; P, splice junction mutation; M, missense mutation; S, synonymous mutation;

Table 3 Mutation sites and mutation types related to the PLEC gene

Case	Reference (year)	Mutation	Protein change	Position	Mutation type
5	Lichtenstein(7), 2012	c.10251del1051ins21	PTC	exon32	F
13	Charlesworth(15), 2013	c.3342-2A>G	--	exon26	P
		c.3902_3903del	PTC	exon28	N
14	Charlesworth(15), 2013	c.4119_4120del	PTC	exon30	F
		c.12499 C>T	p.R4167X	exon32	N
32	Walker(26), 2017	c.2888dupT	PTC	exon23	F
		--	p.Gln2367X	exon31	N

PTC, premature termination codon; F, frame-shift mutation; N, nonsense mutation; P, splice junction mutation; M, missense mutation
